# Development and Validation of the SCAN-Score to Indicate General Anesthesia for Dental Care in Children

**DOI:** 10.3390/jcm13061640

**Published:** 2024-03-13

**Authors:** Pierre-Jean Berat, Romain Jacq, Chloé Villain, Sibylle Vital, Alice Germa

**Affiliations:** 1 Universite Paris Cite, Pediatric Dentistry, DMU ESPRIT, Service de Medecine Bucco-Dentaire, AP-HP, Hopital Louis Mourier, 92700 Colombes, France; sibylle.vital@u-paris.fr; 2 Odontology Department, UFR Health, University of Rouen Nomandy, 76000 Rouen, France; romain.jacq@univ-rouen.fr; 3 DMU ESPRIT, Service de Medecine Bucco-Dentaire, AP-HP, Hopital Louis Mourier, 92700 Colombes, France; 4 Universite Paris Cite, Laboratory Orofacial Pathologies, Imaging and Biotherapies URP 2496, FHU-DDS-Net, 92120 Montrouge, France; 5 Universite Paris Cite, Epidemiology and Statistics Research Center/CRESS, INSERM, INRA, 75004 Paris, France; alice.germa@u-paris.fr; 6 Department of Odontology, APHP, Charles Foix Hospital, 94200 Ivry-sur-Seine, France

**Keywords:** general anesthesia, pediatric dentistry, tooth decay, score

## Abstract

**Background**: General anesthesia is an approach used to address behavior issues in pediatric dentistry. This indication often relies on the practitioner’s discretion rather than objective criteria. We developed SCAN-score to assist pediatric dentists in the case of doubt to indicate general anesthesia for uncooperative children. The study aims were to assess the validity of the SCAN-score, which aims to indicate general anesthesia or chairside management for dental care. **Methods**: A retrospective study was performed on children aged between 2 and 10 years who received dental care. The SCAN-score combined three item scales (age, need of care, behavior) and three additional factors: communication barriers, permanent teeth caries, and local anesthesia contraindications. Mean scores were estimated. An ROC curve was constructed with sensitivities and specificities obtained. **Results**: The study included 284 children, with 154 treated through chairside methods and 130 treated under general anesthesia. The mean score was 5.6 ± 2.8 in the chairside management group and 12.9 ± 1.9 in the general anesthesia group. The sensitivity of the score (cutoff at 10) was 0.99, and the specificity was 0.94. The estimate of the ROC is 0.994. **Conclusions**: The SCAN-score appears to be an excellent tool to support the practitioner’s decision to refer to general anesthesia care.

## 1. Introduction

Childhood dental caries is one of the most common preventable diseases [1]. This multifactorial disease results from the fermentation of dietary carbohydrates by cariogenic bacteria [1]. By 2010, untreated primary tooth decay had affected 9% of the world’s population, amounting to a staggering 621 million children worldwide [2]. Despite the wide range of preventive measures and treatments available, childhood dental caries remains a major health concern and a serious challenge [3].

For the majority of children, dental caries treatment can be effectively managed using behavioral techniques to facilitate communication and reduce undesirable behavior [4,5,6,7]. However, for young children and children with special health care needs (SHCNs), coping with dental care can be particularly challenging.

Special needs are defined by the American Academy of Pediatric Dentistry (AAPD) as “any physical, developmental, mental, sensory, behavioral, cognitive, or emotional impairment or limiting condition that requires medical management, health care intervention, and/or use of specialized services or programs” [8]. Both young age and SHCNs are associated with an increase in dental fear and anxiety, which is an excessive and inappropriate negative emotional state experienced by dental patients [9]. The prevalence rates of dental fear and anxiety vary from 6 to 30%, depending on the scales used, the gender and age of the children or the respondent (parent or child), and represent a major common problem worldwide [10,11,12,13,14]. In severe cases, uncooperative behavior prevents the provision of oral health care, forcing the postponement of treatment and the use of sedation or general anesthesia [4]. Sedation is not always able to provide optimal conditions for the delivery of dental procedures, necessitating the use of general anesthesia [15]. General anesthesia usage is governed by pediatric dental societies, and national organizations have issued guidelines on the indications and contraindications of general anesthesia [4,16]. Indications depend on (1) the child’s general health status, either making chairside care impossible or leading to heavy and urgent oral preparation, related to an organ transplant or cancer, for instance; (2) the nature of the procedure, which might be long and complex, involving several/multiple procedures, or an infectious condition requiring urgent surgical action; and (3) the risks associated with the use of local anesthesia, such as allergies or repeated ineffectiveness.

Despite these guidelines, none of these indications are absolute, leading to considerable variability in practice. Various factors influence decision-making, including practitioners’ perceptions of general anesthesia, their experience, patient cooperation, and accessibility to an operating room [17]. The final decision to indicate general anesthesia appears most often as a personal choice of the practitioner and is not based on objective and reproducible criteria [17,18,19,20,21,22,23]. Moreover, disparities in geographical access to general anesthesia impact patient care [22,24,25].

However, the appropriate utilization of general anesthesia in children is a major issue for both the child and society. Although general anesthesia use is considered relatively safe, it carries a risk of significant morbidity and exceptionally mortality, making its use for patients/practitioner convenience unacceptable [4]. In addition, because the need exceeds the capacity of the operating room, waiting times are substantial, with adverse effects on pediatric patients [24]. Finally, particularly for a preventable disease, general anesthesia has a significant financial cost to the child’s family and to society [19,26,27].

Several authors advocate for the establishment of objective selection criteria or decision trees for dental treatment under general anesthesia [19,28,29,30]. These objective criteria would aim to reduce the variations in indications, despite the presence of guidelines. This should decrease waiting times for patients requiring general anesthesia by directing children to appropriate care, general anesthesia, or specialist chairside care.

In the literature, two scores have been described. The first score, proposed by The Texas Medicaid & Healthcare Partnership, an insurance company, aimed to facilitate payment approval for general anesthesia [31]. Criteria used were the patient’s age, treatment requirements, behavior, need for surgical intervention, previous failed sedation, medical conditions, and disabilities. However, this score’s limitation lies in its lack of scientific evidence [31]. The second score was intended to direct children either to chairside dental care combined with midazolam or to dental care under general anesthesia [29]. Regrettably, there exist notable international disparities regarding dentists’ access to deep sedation [32]. Often, practitioners face a choice between chairside management, which may include conscious sedation, and general anesthesia.

Consequently, a scoring tool was devised by the Department of Pediatric Dentistry at Louis Mourier Hospital—Universite Paris Cite (Colombes-APHP, France), which is a referral center for pediatric dental care when it cannot be carried out conventionally in the community. The SCAN-score (Score based on Child’s behavior, Age and Need) has been developed to help pediatric dentists assess key factors in uncooperative children, when deciding on the most appropriate treatment, particularly whether or not general anesthesia is indicated.

The objective of this study was to assess the validity of the SCAN-score, which aims to streamline the decision-making process for referral to either general anesthesia or chairside management with or without sedation.

## 2. Materials and Methods

### 2.1. SCAN-Score

The SCAN-score developed had 3 main items: (1) age, (2) need for care, and (3) children’s behavior, and 3 additional points: communication barriers (related to SHCNs or foreign language), permanent tooth requiring treatment, and contraindications to local anesthesia (Table 1). The need for care was determined as the number of sextants requiring treatment, i.e., the number of appointments needed in the chairside management alternative. Teeth that required only fissure sealants or fluoride varnish were not considered as “requiring treatment”. Behavioral items assess dental fear and anxiety and uncooperative behavior during the first consultation or first dental treatment. This assessment was partly based on the Venham scale modified by Veerkamp [33]. It was assessed as reluctance to care, acceptance of the clinical examination (±intraoral radiograph) but refusal to perform care, or absence of cooperation for the clinical examination.

Each item and potential bonuses had a value; the sum corresponded to a total score. Need for general anesthesia was defined by a total score greater or equal to 10. The score was developed retrospectively and was not used to refer patients to chairside management or general anesthesia.

The SCAN-score was retrospectively calculated by reviewing the records of each included patient.

### 2.2. Population

This retrospective study included children aged 2 to 10 years who received dental care at the Pediatric Dentistry Department of Louis Mourier Hospital, AP-HP, Universite Paris Cite (Colombes, France), from January 2018 to December 2019. To be included in the study, children must have an ASA score of 1 or 2, French health insurance coverage, and at least one dental treatment session performed in the department either under general anesthesia or with chairside management. ASA refers to the physical status classification system of the American Society of Anesthesiologists. ASA 1 is a normal healthy patient, and ASA 2 is a patient with mild systemic disease without significant functional limitations [34]. The age limit was set at 10 years, as older children undergoing dental general anesthesia typically have clear indications, primarily related to health issues [22]. For regulatory reasons, children under the age of 2 cannot receive dental treatment under general anesthesia at our clinic. In addition, children must have a body weight of more than 10 kg.

All children were treated by a senior practitioner trained in pediatric dentistry. General anesthesia was performed by an anesthesiologist team, using sevoflurane, IV propofol, and muscle-relaxing drugs. Chairside management may have been associated with hydroxyzine administration and/or nitrous oxide. Treatments involved direct restorations, stainless steel crowns, extractions, pulpotomies, and endodontic treatments. Under general anesthesia, the whole treatment plan was performed in one session, whereas chairside management required between one and six sessions.

### 2.3. Data

Data were collected from patient records, including demographic characteristics (age, sex), health conditions (medical and surgical history, current medication, intended treatments), dental needs (number of sextants to be treated), and behavior during the chairside clinical examination, including the ability to undergo radiographs. The outcome was the orientation between chairside management and general anesthesia. The children, initially addressed for chairside management, who finally required general anesthesia were considered as having general anesthesia. The success or failure of the sessions was also recorded. Session failure was defined as a session in which the child’s cooperation could not be obtained or physical restraint was required to carry out treatment.

### 2.4. Statistical Analysis

The mean total scores (and standard deviations) and the mean of each item (and standard deviation) subscores were estimated.

Total score and each subscore in the general anesthesia group and in the chairside management group were compared using the *t*-test. The sex ratio, the frequency of care on permanent teeth, and the frequency of contraindications to local anesthesia in the two groups were compared by chi-square test.

The significance level was determined as *p*-value < 0.05.

A receiver operating characteristic curve (ROC curve) was constructed with sensitivities and specificities obtained at all possible total scores. Sensitivities and specificities at all possible scores were compared to determine the optimal cut-off value of 10 for distinguishing between general anesthesia and chairside management.

Analyses were performed with R version 4.3.1 (2023-06-16)—“Beagle Scouts” © 2023 software.

## 3. Results

Of the 306 children aged 10 or younger who had consultations in the Department of Pediatric Dentistry at Louis Mourier Hospital, 284 were included in this study after excluding incomplete records. The mean age of the children was 5.9 years (SD = 2.2), and 58% of them were male (Table 2). Almost half of the 33 children with special needs had autism spectrum disorders, while 4 children spoke a language other than French or English. The remaining children with special needs had learning or speech disabilities, Down syndrome, or other medical conditions. General anesthesia was indicated for 130 children, while 154 children were scheduled for chairside management.

The significantly higher mean total score observed in the general anesthesia group (12.9 ± 1.9) compared to the chairside management group (5.6 ± 2.8) underscores the effectiveness of the SCAN-score in stratifying patients based on their suitability for different treatment modalities. The broader range of scores observed in the general anesthesia group (8 to 17) compared to the chairside management group (0 to 11) highlights the heterogeneity in treatment needs and behavior profiles among pediatric dental patients, further emphasizing the importance of individualized care planning.

Children treated under general anesthesia had significantly higher scores (*p < 0.001*), were significantly younger (*p < 0.001*), with a greater need for treatment (*p < 0.001*), and had more behavior problems (*p < 0.001)* than those treated with chairside management. There was no significant difference in the frequency of care on permanent teeth. Additionally, no children had any contraindications to local anesthesia (Table 1).

Among the 130 children in the general anesthesia group, only 1 had a score less than 10 (score of 8). Among the 154 children in the chairside management group, 9 had a score greater than or equal to 10.

**Figure 1:**
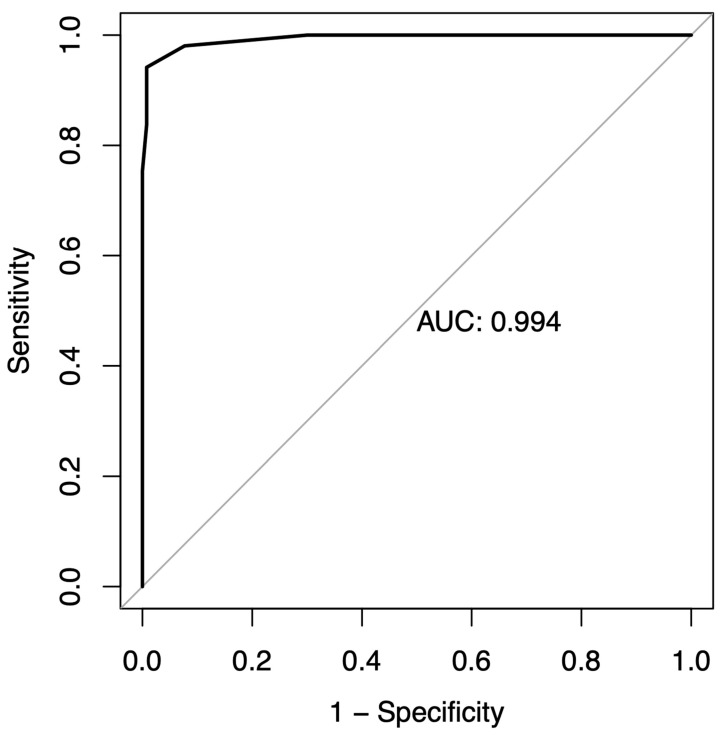
Receiver operating characteristic (ROC) curve.

Score has a sensitivity of 0.99 and a specificity of 0.94 (Table 3), and the Receiver Operating Charactristic (ROC) curve displays an Area Under the Curve (AUC) value of (Figure 1).

## 4. Discussion

We report a concise and straightforward assessment tool for pediatric dentists to determine whether uncooperative children should be referred to general anesthesia or chairside management when the question arises. With a sensitivity of 0.99 and specificity of 0.94, the SCAN-score effectively discriminated between children requiring general anesthesia and those who can be managed chairside, with or without sedation. These findings are supported by the large and diverse sample size used in this study, involving 284 participants divided into two well-balanced groups, enhancing the reliability of our results.

The proposed score offers a succinct summary of the essential criteria for deciding the need for general anesthesia, in accordance with the established professional guidelines [4,16]. This easy-to-use instrument, with a threshold set at 10, relies on universally acknowledged criteria or validated behavioral assessment scales, which improve its reproducibility.

Firstly, the age of the child is considered, with maximum weight given to children up to 3 years old, and decreasing weights assigned with increasing age up to 7 years. Indeed, as children grow, they typically develop the ability to handle dental care, especially in the absence of additional conditions [10]. The effectiveness of behavior management techniques is influenced by the child’s age and level of understanding [35,36,37]. In addition, a child’s dental fear and anxiety can directly influence their behavior during dental procedures [36], with the prevalence of these fears decreasing with age [13].

Secondly, the need for care was assessed by sextant. It is established that children undergoing dental treatment under general anesthesia require more extensive care compared to children receiving chairside dental care [18,29,35]. They have not only more decayed teeth but also require more invasive care, such as pulpotomies, stainless steel crowns, and avulsions [30,36]. We made the decision not to incorporate the type or complexity of care as an item in the SCAN-score due to the difficulties practitioners encounter in conducting a thorough clinical assessment during the initial consultation, especially regarding X-rays when a child is uncooperative. Thus, the “need for care” item was defined based on the number of sextants requiring treatment, a criterion easily applicable to all children and minimizing the risk of misclassification.

Then, the behavioral assessment methodology was grounded in the Venham Scale, which is a rigorously validated metric in the extant literature [13,14]. The Venham scale can easily be replaced by the Frankl Scale [37] or the Modified Child Dental Anxiety Scale [38] in the SCAN-score to adapt to different practice habits [14,38].

Three additional points were incorporated into the SCAN-score. Firstly, consideration of cavities in permanent teeth was included, as their presence can complicate treatment and prolong sessions. Secondly, communication barriers, such as those related to SHCNs or language differences, were taken into account. It is well established that the success of dental treatment hinges largely on a child’s ability to manage dental stimuli effectively [36,38]. Consequently, uncooperative children, particularly those with SHCNs like autism spectrum disorders or other neurodevelopmental disorders, are more frequently referred to dental treatment under general anesthesia [35,39]. However, not all children with SHCNs or those who are uncooperative require general anesthesia for treatment. Building cooperation and adaptation of the dental office is essential, and pediatric dentists play a crucial role in enhancing children’s cooperation and adaptation [40]. Non-pharmacological techniques can be utilized to improve a child’s cooperation at the dental office, such as creating a multisensory-adapted dental environment in the waiting room to reduce pre-appointment dental anxiety in children with developmental disabilities [41], or employing dog-assisted therapy to enhance acceptance during dental examinations and procedures [7].

Lastly, in accordance with the guidelines, the consideration of contraindications to the use of local anesthetics was included in the SCAN-score [4,16], although such contraindications remain extremely rare.

Other assessment tools have been developed and applied in the literature, including the scoring system devised by Mohan et al., which comprises six items and utilizes a threshold of 18 [29,42,43]. Mohan’s score serves a distinct purpose, focusing on distinguishing between the need for care under general anesthesia versus sedation with midazolam. We opted to compare general anesthesia with chairside management, including or excluding nitrous oxide sedation. While many studies have compared general anesthesia with nitrous oxide and oxygen inhalation sedation [44], the availability of midazolam for dental procedures varies widely. In France, its use is quite limited to dental care, with only nitrous oxide sedation being available in our clinic.

As for Prabhu et al., they proposed a comprehensive screening tool that integrates patients’ behavior, potential medical risks associated with treatment, their level of cooperation and communication, as well as consent, the availability of facilities for general anesthesia, and the type of dental treatment planned [45]. The objectives of the tool developed by the authors differ from ours as it focuses only on patients with disabilities. For patients with severe pathologies, the anesthetic risk becomes a critical factor in deciding whether to recommend general anesthesia, making the SCAN-score unsuitable for such cases. Moreover, it is also intended for adults, with specific problems, such as obtaining consent. Finally, it is intended to guide clinicians who are inexperienced in the referral process, in contrast to our score.

The SCAN-score could be tailored to suit specific contexts, particularly taking into account the availability of alternative sedation methods. For example, the threshold for general anesthesia, currently set at 10, could be adjusted upwards. Children falling between 10 and this new threshold could then be referred for treatment under deep sedation as an alternative approach.

The criterion of long-distance travel to reach the clinic, although previously recommended as a factor favoring general anesthesia [22], was not included in the SCAN-score. This decision was based on the context of our clinic, situated in a densely populated urban area where long-distance travel is not a prominent concern. However, it is acknowledged that this factor may be pertinent for other teams, and thus the SCAN-score can be adjusted to accommodate such considerations.

This study has three main limitations. Firstly, its single-center design introduces inherent confirmation bias, especially considering that the SCAN-score was developed within our specific context. Consequently, modifications may be necessary to adapt the score to different settings with the varying availability of deep sedation and relevance of travel distance, among other factors. Additionally, it should be noted that the score may not be suitable for medical emergency situations where immediate prognosis is crucial.

Secondly, the retrospective study design may lead to limited or missing data during data collection. Thirdly, medically compromised children were not included in the study, as our clinics primarily treat children classified as ASA 1 or ASA 2. This exclusion may impact the generalizability of our findings to broader populations.

Lastly, the study population comprised only children referred to our pediatric department following management failure, thus representing a specialized group with specific needs. Nevertheless, our population closely mirrors those in other studies on pediatric dentistry under general anesthesia, with an average age of 4.9, as compared to 4.7 [46,47]. These characteristics precisely reflect the population where the decision to use general anesthesia is debatable, making them the ideal population to evaluate the SCAN-score.

However, the aim of the SCAN-score is not to escalate the number of children undergoing dental procedures under general anesthesia. Rather, it serves as a tool for pediatric dentists who have received appropriate training and possess the necessary skills to deliver comprehensive care in reluctant children [41]. It is worth noting that in numerous cases, children initially referred to general anesthesia by a general dentist can be successfully managed chairside [23,25].

## 5. Conclusions

The SCAN-score appears as an easy-to-use tool, devised to assist the pediatric dentist in referring children for dental general anesthesia or chairside management, especially when in doubt. The SCAN-score is particularly recommended for pre-school children with early childhood caries. It offers a solution to the lack of objective criteria for making decisions to refer to general anesthesia. Its simplicity enables it to be adapted to local practices related to the care of children and should be studied in other contexts. Furthermore, this tool could be very useful in communication with anesthesiologists. However, the SCAN-score shall not surrogate the clinical judgment and assessment of the dental surgeon to validate the indication of general anesthesia.

## Figures and Tables

**Table 1:** Scale to assess the need for general anesthesia.

	Score
Age	
6–7 years	1
5 years	2
4 years	3
3 years and under	4
Need for care	
1 sextant	1
2 sextants	2
3 sextants	4
4 sextants and more	5
Behavior	
Dental anxiety and uncooperative behavior, assessed as reluctant during care	3
Acceptation of the clinical examination (± intraoral radiograph) but refusal to perform care	5
Absence of cooperation for the clinical examination	6
Bonus	
Communication barriers (related to SHCNs or foreign language)	3
Care on permanent teeth needed	1
Contraindication to local anesthesia	9
Total	=

**Table 2:** Description of the score in the general population, in the chairside management group, and in the general anesthesia group.

	General Population		Chairside Management		General Anesthesia		
	N/Mean	%/sd	N/Mean	%/sd	N/Mean	%/sd	*p*-Value ^a^
Total	284		154	54%	130	46%	
Sex							
male	164	58%	88	57%	76	59%	*0.82*
Age (year)	5.9	±2.2	6.7	±2.1	4.9	±1.8	*<0.001* *
Need for care	3.9	±1.8	3.1	±2.0	4.9	±0.6	*<0.001* *
Behavior	2.6	±2.3	0.8	±1.4	4.7	±1.0	*<0.001* *
Bonuses	0.2	±0.4	0.2	±0.4	0.1	±0.3	*0.1*
Communication	33	11.6%	4	1.4%	29	10.2%	*<0.001* *
Care on permanent teeth needed	51	18%	33	12%	18	6%	*0.1*
Contraindication to local anesthesia	0	0%	0	0%	0	0%	*-*
Total score	8.9	±4.3	5.6	±2.8	12.9	±1.9	*<0.001* *

^a^ chi-square *p*-value for categorical variables or *t*-test *p*-value for continuous variable. * *p* < 0.05.

**Table 3:** Score distribution according to the cutoff value of 10 in the chairside management group and in the general anesthesia group.

	Number of Children		
	Score ≥ 10	Score < 10	Total
General anesthesia	129	1	130
Chairside management	9	145	154
Total	138	146	284

## Data Availability

The raw data supporting the conclusions of this article will be made available by the authors on request.

## Abbreviations

SHCNs: special health care needs.
